# [Retracted] MicroRNA-486-5p inhibits the growth of human hypertrophic scar fibroblasts by regulating Smad2 expression

**DOI:** 10.3892/mmr.2026.13831

**Published:** 2026-02-25

**Authors:** Yingying Shi, Luping Wang, Pijun Yu, Yi Liu, Wei Chen

Mol Med Rep 19: 5203–5210, 2019; DOI: 10.3892/mmr.2019.10186

Following the publication of the above paper, it was drawn to the Editor's attention by a concerned reader that certain of the flow cytometric data shown in Fig. 5A on p. 5208 were strikingly similar to data that had appeared previously in other papers written by different authors at different research institutes.

In view of the fact that the abovementioned data had already apparently been published prior to its submission to *Molecular Medicine Reports*, the Editor has decided that this paper should be retracted from the Journal. The authors were asked for an explanation to account for these concerns, but the Editorial Office did not receive a reply. The Editor apologizes to the readership for any inconvenience caused.

